# Protocol for a multicenter observational prospective study of functional recovery from stroke beyond inpatient rehabilitation - The Interdisciplinary Platform for Rehabilitation Research and Innovative Care of Stroke Patients (IMPROVE)

**DOI:** 10.1186/s42466-020-00056-2

**Published:** 2020-04-06

**Authors:** Gunnar Birke, Silke Wolf, Thies Ingwersen, Christian Bartling, Gabriele Bender, Alfons Meyer, Achim Nolte, Katharina Ottes, Oliver Pade, Martin Peller, Jochen Steinmetz, Christian Gerloff, Götz Thomalla

**Affiliations:** 1 Department of Neurology, University Medical Centre Hamburg-Eppendorf, Martinistr. 52, 20246 Hamburg, Germany; 2 MediClin Klinikum Soltau, Oeninger Weg 59, 29614 Soltau, Germany; 3 RehaCentrum Hamburg GmbH, Martinistraße 66, 20246 Hamburg, Germany; 4 VAMED Klinik Geesthacht, Johannes-Ritter-Straße 100, 21502 Geesthacht, Germany; 5 Klinikum Bad Bramstedt, Klinik für Neurologische Rehabilitation, Oskar-Alexander-Straße 26, 24576 Bad Bramstedt, Germany; 6 VAMED Rehaklinik Damp, Seute-Deern-Ring 30, 24351 Damp, Germany

**Keywords:** Stroke, Rehabilitation, Functional recovery, Hand motor function, International classification of functioning, Disability and health

## Abstract

**Introduction:**

Stroke and its long-term consequences pose major challenges for the lives of those affected and healthcare systems. Neurological rehabilitation therefore primarily attempts to improve function in order to increase independence in activities of daily living, and to enable social participation. There is only scarce data on dynamics of functional recovery after patients discharge from inpatient neurological rehabilitation. Even less is known about the patient’s perspective on long-term recovery from stroke. The Interdisciplinary Platform for Rehabilitation Research and Innovative Care of Stroke Patients (IMPROVE) aims to address this knowledge gap by providing new insights into the dynamics and extent of functional recovery from stroke beyond inpatient rehabilitation treatment.

**Methods:**

We provide the protocol for an observational, longitudinal, multicenter study conducted in an Universitary Stroke Center in cooperation with five Neurological Rehabilitation Centers in Northern Germany. Patients who suffered from ischemic or hemorrhagic stroke will be enrolled by the end of inpatient rehabilitation and followed up to 1 year. In addition, a group of chronic stroke patients and a group of craniocerebral trauma patients will be enrolled as a comparison group. Data on stroke characteristics, vascular risk factors, co-morbidities, social support, and demographics will be recorded. Comprehensive clinical evaluation will be performed at baseline, three, six, and twelve months after enrollment. The assessments and scores used reflect the three components of the International Classification of Functioning, Disability and Health (ICF), some of them are tests regularly used in rehabilitation settings. Tests of motor function, cognition, and mood are included, as are tests of self-reported health-related quality of life. Primary outcome measure is a hand motor score, built by the sum of the hand items of the Fugl-Meyer Assessment as an objective measurement of hand function at 12 months after enrollment. Predictors of the primary outcome will be analyzed using linear regression analysis.

**Perspective:**

The results of IMPROVE will inform about the long-term dynamics of functional stroke recovery after patients’ discharge from inpatient rehabilitation and will provide insights into the association of clinical and demographic factors with recovery of function.

**Trial registration:**

The protocol is registered at ClinicalTrials.gov (NCT04119479).

## Introduction

Cerebrovascular diseases, such as stroke, are among the greatest challenges in healthcare [23, 24]. In Germany, about 196,000 first-ever strokes occur every year. In addition, about 66,000 recurrent strokes take place [21]. In the context of the demographic development with an ageing population, the number of stroke patients is expected to increase significantly in the coming years [14]. In parallel with the increasing incidence and decreasing mortality, however, it is above all the impairments caused by a stroke that are of great interest to health care practitioners and other health care actors [33, 41].

Stroke, the second leading cause of death worldwide, represents one of the main reasons for long-term disability [45]. In high-income countries, stroke is even the most common cause of acquired impairment in adulthood, and some of those affected are confronted with dramatic changes in patients’ everyday lives [17, 43]. Data on long-term disability and recovery after stroke vary widely and depend on various influencing factors, like age or type of stroke [6, 46]. However, motor impairments after stroke are found in about 80% of all cases [27], which are associated with persisting disability and dependency in over 30% [12].

The importance of neuro-rehabilitative research becomes obvious in regard to these numbers [5]. The main aim of neurological rehabilitation is to improve function and independence in activities of daily living, and to enable social participation [12]. The understanding of rehabilitation must not only be based on the underlying disease mechanisms, but must above all be based on the consequences of the disease according to the International Classification of Functioning, Disability and Health [44]. This concept divides the disease consequences into three main areas: (a) damage to body functions and structure (e.g. paresis of the hand), (b) activity (e.g. learning activities, exercise of skills) and (c) participation (e.g. participation in professional and social life). In addition to pathophysiological classification, this system offers the possibility of classifying and specifically treating patients with regard to their functional state of health, the degree of limitation and their social effects.

Stroke research is often focused on the acute treatment phase as well as on the first weeks of rehabilitation – usually the phase of inpatient rehabilitation [40]. Data on long-term disability and recovery after stroke is limited. Few studies have explored functionality based on the ICF conceptual model [38].

In stroke patients, a wide range of individual variability of recovery extent and rate can be observed clinically. However, it is still open which factors contribute to functional recovery. Detailed data describing the long-term development of stroke patients after being discharged from rehabilitation programs are rare. To date, it is not fully described how stable the effects of rehabilitation are and how much clinical improvement can be seen in the time period following inpatient rehabilitation, especially with regard to ICF functionality. Even less is known about the patients’ perspective on long-term recovery from stroke.

This lack of data is also due to a missing link between research institutions and rehabilitation centers [19, 39]. Therefore, the IMPROVE-platform was established as a research collaboration between an Universitary Stroke Center, i.e., the department of neurology at the University Medical Center Hamburg-Eppendorf (UKE) and five rehabilitation clinics, to address the mentioned questions in future research projects.

This first study of the IMPROVE platform is a multicenter observational prospective study to investigate the main factors influencing the functional recovery of stroke patients with regard to the ICF-dimensions in a long-term view with a focus on hand function.

## Methods

### Aim of the study

The overall goal of this study is to identify and understand factors that influence the course of recovery following focal brain lesions within the framework of standard neurorehabilitation in Germany. In this observational longitudinal study, the long-term recovery processes of stroke patients will be investigated. The influence of motor function, cognition, care situation, depression, fatigue as well as information and knowledge about the disease on functional recovery, participation, autonomy and quality of life will be assessed. The assessment of motor function focuses on hand-function, which represents a crucial function for independence and participation in activities of daily living. The current state of subjective as well as objective parameters is surveyed. The development of hand function 1 year after rehabilitation and the main factors influencing it will be analyzed. Ideally, the results obtained can be used to identify opportunities and improvement potentials for future rehabilitation strategies. More targeted planning and individualized therapy courses could be results of this development.

### Study description and study design

**Fig. 1:**
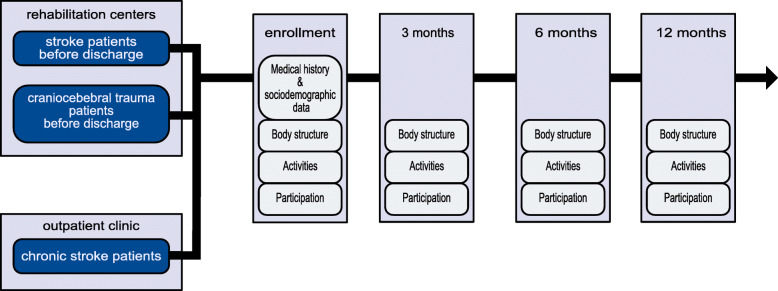
Stroke patients with an ischemic or hemorrhagic stroke with a still remaining deficit (mRS ≥1) during the end of inpatient rehabilitation are included in the study. In addition, a group of patients with craniocerebral trauma from the rehabilitation centers as well as a group of chronic stroke patients is recruited as a comparison group. All groups undergo the same examinations and ICF-orientated assessments

IMPROVE is an observational, longitudinal, multicenter study in an Universitary Stroke Center and five rehabilitation centers in northern Germany (NCT04119479). The applied set of assessments includes self-reported outcome measures as well as objective scores representing the ICF dimensions body function and structure, activity and participation. Initial measures are conducted during the end of inpatient rehabilitation in the cooperating study centers. Follow-up examinations take place three, six and 12 months after inclusion (Fig. 1). Measures include standardized state of the art motor and cognitive function assessments carried out by trained health care specialists. In addition, questionnaires are provided covering a number of stroke specific health care topics. If available, routine magnetic resonance imaging data from the acute care phase is collected as well. Before inclusion, patients are informed thoroughly about the study structure and the ability to opt-out at any given point. Data collection is carried out according to European data protection law.

### The IMPROVE research platform

For this research project, rehabilitation centers suitable for implementation were sought and approached. The general study structure was discussed in preparatory meetings and developed together with the participating centers. In the first months of 2017, the last adjustments to the study design were made, particularly with regard to the selection of assessors.

For the implementation in clinical everyday life of the rehabilitation clinics, an intensive exchange with the cooperation clinics took place via visits and various meetings. Task force meetings are hold, which are attended by one representative of each of the partner clinics. In addition, joint cooperation meetings involving the local medical study management and representatives of the therapy areas of all clinics are performed. At the same time, there are regular telephone and E-mail exchanges, covering individual questions and assessment contents. A monthly IMPROVE newsletter is set up to inform all participants about current activities and the current recruitment status.

An initial standardized on-site training was given to the staff in each of the participating rehabilitation clinics, covering the topics inclusion, documentation, the exact performance of assessments and the exchange of data between the rehabilitation clinic and UKE. Only trained personnel are allowed to carry out the study in order to ensure a standardized implementation. A test run of one to two patients per clinic was initially carried out for quality assurance and in order to integrate the study into the processes of the clinics. If new members join the study team, they are also trained in the IMPROVE standards. An electronic Case Report Form is set up for the IMPROVE study. All follow-up examinations are carried out at the UKE by the same trained study team. In order to enable all patients to participate, travel costs are reimbursed or transport to the study center is organized.

### Study arms

Three groups are included in the study. Mainly subacute stroke patients with an ischemic or hemorrhagic stroke at the end of their inpatient rehabilitation period. Only patients with a still remaining deficit are included. In one rehabilitation center, craniocerebral trauma patients at the end of the inpatient rehabilitation are recruited as a second group. Additionally, a cohort of chronic stroke patients is recruited by the post stroke clinic of the UKE Neurology department. The chronic stroke group as well as the group of craniocerebral trauma patients represent a comparison group. All groups undergo the same examinations.

### Eligibility criteria

Patients admitted to the cooperating rehabilitation centers following cerebral infarction and/or cerebral hemorrhage (ICD 10 I61-I69) are screened by a physician for eligibility. Patients from the rehabilitation centers are included at the end of rehabilitation phases C and D according to the criteria of the Bundesarbeitsgemeinschaft für Rehabilitation [3].

Inclusion criteria are:

- Ischemic or hemorrhagic stroke according to ICD 10 I61-I69
- Patients in or after completion of rehabilitation phases C and D according to BAR criteria
- Age ≥  18
- Sufficient knowledge of German
- Existing declaration of consent
- Deficit still existing (modified Rankin score of at least 1 at inclusion)

Exclusion criteria are:

- Need for care prior stroke
- Subarachnoid hemorrhage, craniocerebral trauma, transient ischemic attack as primary diagnosis
- Severe pre-existing psychiatric disease
- Participation in follow-up examination not possible

For the chronic stroke group as well as the craniocerebral trauma group the same inclusion/exclusion criteria are applied. For the chronic stroke group patients are included in an outpatient setting after completing inpatient rehabilitation. For the craniocerebral trauma group patients with craniocerebral trauma (ICD 10 S06.1-S06.9) as a primary diagnosis are included.

### Sample size estimation

As this is an explorative observational study, no classical calculation of sample sizes is eligible. Based on the annual treatment numbers of the participating clinics, an initial estimate of 300 inclusions was made. Eighteen months after the beginning of the study we performed a sample size calculation based on the available data on recovery of the hand function in the Stroke Impact Scale (SIS) and the dropout rate of the patients included until then. Based on these data we estimated that we would need *N* = 225 to be able to identify a difference with Cohens d = 0.3. The recruitment goal could be reduced to 225 patients.

### Outcome measures

The primary outcome measure is a hand motor score built by the sum of the hand items of the Fugl-Meyer upper limb motor score as an objective measurement of hand function after 1 year [15]. This subdomain covers seven performance tasks rated in a three-point scale from 0 = cannot perform, 1 = performs partially and 2 = performs fully. The seven tasks include Finger flexion (1), Finger extension (2), Extension of metocarpophalangeal joints, flexion of proximal and distal interphalangeal joints (3), Thumb adduction (4), Thumb opposition (5), Grasp cylinder (6), Grasp tennis ball (7). The maximal possible score is 14.

Further outcome measures include assessments and scores that are regularly used in rehabilitation settings and are designed to reflect the three components of the International Classification of Functioning, Disability and Health (Tables 1, 2 and 3).

**Table 1:** Assessments used in the study reflecting the component body structure of the International Classification of Functioning, Disability and Health

Body structure	
National Institutes of Health Stroke Scale	The National Institute of Health Stroke Scale is a score system to quantify the impairment caused by a stroke. The sum of the values from the investigations results in a maximum of 42 points. The higher the score, the more extensive the stroke [7].
Fugl-Meyer Assessment (upper extremity)	The section motor function of upper limb is one of five domains, a three-point scale is used for rating performance as 0 = cannot perform, 1 = performs partially and 2 = performs fully, maximal possible score: 66 points [15].
Grip and pinch strength	A dynamometer is used to measure grip strength and a pinch gauge to measure pinch force.
Montreal Cognitive Assessment	The Montreal Cognitive Assessment is a screening assessment for detecting cognitive impairment, a maximum of 30 points (no restrictions) can be achieved [30].
Line Bisection Test	The line bisection test is a test to detect the presence of unilateral spatial neglect. To complete the test, the middle of several horizontal lines is estimated and marked [1, 2].
Bells Test	The Bells test is a cancellation task used for quantitative and qualitative evaluation of visual neglect. Patients are asked to find bells that are distributed pseudo-randomly among distractive stimuli [16].
Aphasia Test	Standardized test for differential diagnosis Aphasia - no aphasia [26].
Apraxia Screen of TULIA	The Apraxia Screen from TULIA is a short assessment to diagnose apraxia with 12 hand movements, dichotomous scale: 0 = not fulfilled, 1 = fulfilled motion task based on the comprehensive standardized Test for Upper-Limp Apraxia (TULIA) [42].
ASKU self-efficacy short form	A 3-items scale for the measurement of self-efficacy [4].

**Table 2:** Assessments used in the study reflecting the component activities of the International Classification of Functioning, Disability and Health

Activities	
modified Rankin Scale	The modified Rankin scale (mRS) is a standardized measure that describes the extent of disability after a stroke. It ranges from 0 = no symptoms to 6 = death due to stroke [8, 34].
Timed Up and Go Test	The Timed “Up and Go” test is a clinical test to assess a patient’s mobility and risk of falling [32].
Nine Hole Peg Test	The Nine Hole Peg Test is a timed measure of fine manual dexterity where the patient is instructed to first take nine pegs out of a container and subsequently place them back into the empty holes of the container as quickly as possible [29].
Barthel Index	The Barthel Index is an ordinal scale that accounts for the patient’s autonomy and need for care. It covers essential activities of daily living and ranges from 0 (total dependency) to 100 (independent) [37, 28].
Fatigue scale for motor function and cognition	Fatigue scale for motor and cognitive functions, an assessment of fatigue, containing two subscales (mental and physical fatigue), ranging from 20 = no fatigue at all to 100 = severest grade of fatigue [31].
ICHOM-Questionnaire	A standard Set of Patient-reported Outcome Measurements for stroke from The International Consortium for Health Outcomes Measurements including the PROMIS-10 [35].
AUDIT C	The Alcohol Use Disorders Identification Test (AUDIT) alcohol consumption questions. A Screening Test for Problem Drinking [9].
Fagerström Test	A 6-items questionnaire for nicotine dependence [13].

**Table 3:** Assessments used in the study reflecting the components participation of the International Classification of Functioning, Disability and Health

Participation	
Stroke impact scale (SIS)	Measurement of subjective stroke-specific health status, 64 items in eight domains, domain scores range between 0 and 100, with higher scores represent better health status [11]. Since this questionnaire includes questions about stroke impact in the last 4 weeks, it is applicable starting from the first follow-up after 3 months.
Index for measuring restrictions on participation (IMET)	The Index of measurement of participation restrictions (IMET) records patient-related participation as a self-evaluation tool, on a scale from 0 = no impairment to 10 = no more activity possible [10].
Patient Health Questionnaire 4	The Patient Health Questionnaire 4 is a screening tool for diagnosing depression and includes questions on the nine DSM-IV criteria for the diagnosis of major depression [25].
Patient reported health status (EQ-5D)	The EQ-5D questionnaire is a standardized, generic measure of health-related quality of life. It is a self-administered questionnaire [22, 20].
Return to work	A questionnaire developed by the research group (including questions on occupation and lifestyle).
ZAPA	Questionnaire assessing satisfaction with outpatient care with focus on patient participation [36].

In addition, medical history is recorded at each examination time point. Questionnaires about health care structure (ZAPA) [36], level of information and information needs and sociodemographic information [18] are included.

Certain data regarding sociodemographic information and clinical characteristics of stroke are collected at the first examination. Questionnaires which are just applicable for patients, which have left the hospital environment (like the SIS) are added to the assessments starting from the first follow-up after 3 months.

If available, magnetic resonance imaging from the acute phase are acquired and stroke volume and location is analyzed using semi-automatic segmentation software.

### Contacts

The study is conducted as a multicenter study by the Department of Neurology of the University Medical Center Hamburg-Eppendorf in collaboration with the departments for neurological rehabilitation of RehaCentrum Hamburg, MEDICLIN Klinikum Soltau, Klinikum Bad Bramstedt, VAMED Klinik Geesthacht and VAMED Rehaklinik Damp. The study is funded by the University Medical Center Hamburg-Eppendorf and the Deutsche Rentenversicherung Nord.

### Perspective

While stroke rehabilitation research often focuses on the acute treatment phase and the time period of inpatient rehabilitation, we designed a study to investigate the long-term development of stroke patients after discharge from the rehabilitation clinic. Recruitment to the study was started in July 2017. The estimated primary completion date is July 2020.

The data of this study will provide an insight into the long-time course of stroke rehabilitation. The data collected have the potential to identify factors that influence recovery from stroke in the long term. This can be the basis for further treatment improvements in stroke rehabilitation by addressing potentially unknown factors and thus individualize therapy paths.

Besides that, investigating the long-term course of chronic stroke patients will give an important information about the recovery potential of these patients in a standard care setting. Quality of life at the end of the rehabilitation as well as changes in quality of life over 1 year will be investigated. Data on patients’ stroke-related health literacy and information needs over 1 year will be analyzed.

Overall, this study can contribute to a better understanding of the long-term course of stroke patients, thereby help health professionals dealing with stroke with an earlier prognosis and maybe provide data for patient-individualized treatment-protocols in the future.

With this study, the IMPROVE-Platform is established as an interdisciplinary platform for rehabilitation research for stroke patients. This first study addressing the actual practice of neurorehabilitation will be the basis for building new research hypotheses, which than can be tested in future studies within the IMPROVE-Platform. This platform connects experts from research and clinical practice of neurorehabilitation and thereby forms a network to build up future research projects in the area of neurorehabilitation.

## Data Availability

The data will be deposited on a protected server of the University Medical Centre Hamburg-Eppendorf. Access is strongly regulated even for study personnel. Owing to the difficulty of de-identification (routine care, qualitative data, etc.), individual participant data will not be shared publicly. Upon reasonable request that includes a methodologically sound proposal for the usage of data that is also approved by the responsible review committee data may be shared.

## Abbreviations

IMPROVE: The Interdisciplinary Platform for Rehabilitation Research and Innovative Care of Stroke Patients
ICF: International Classification of Functioning, Disability and Health
mRS: Modified Rankin Scale
UKE: University Medical Center Hamburg-Eppendorf
ICD: International Classification of Diseases
BAR: Bundesarbeitsgemeinschaft für Rehabilitation
SIS: The Stroke Impact Scale
ZAPA: Questionnaire assessing satisfaction with outpatient care with focus on patient participation - “Fragebogen zur Zufriedenheit in der ambulanten Versorgung – Schwerpunkt Patientenbeteiligung (ZAPA)”
TULIA: Test for Upper-Limb Apraxia
ASKU: Short scale for the measurement of self-efficacy – “Allgemeine Selbstwirksamkeit Kurzskala”
ICHOM: The International Consortium for Health Outcomes Measurements
PROMIS-10: Patient-reported Outcomes Measurements Information System 10-Question Short Form
AUDIT C: Alcohol Use Disorders Identification Test
SIS: Stroke impact scale
IMET: The Index of measurement of participation restrictions
EQ-5D: European Quality of Life 5 Dimensions
